# Model-Based Analysis of In Vivo Release Data of Levonorgestrel Implants: Projecting Long-Term Systemic Exposure

**DOI:** 10.3390/pharmaceutics15051393

**Published:** 2023-05-02

**Authors:** Soyoung Kim, Brian Cicali, Michelle Pressly, Lais Da Silva, Thomas Wendl, Valvanera Vozmediano, Stephan Schmidt, Rodrigo Cristofoletti

**Affiliations:** 1 Center for Pharmacometrics and Systems Pharmacology, Department of Pharmaceutics, College of Pharmacy, University of Florida, Orlando, FL 32827, USA; 2 Bayer AG Pharmaceuticals, 51377 Leverkusen, Germany

**Keywords:** hormonal contraceptives, subcutaneous implants, PBPK modeling

## Abstract

Levonorgestrel (LNG) is a progestin used in many contraceptive formulations, including subcutaneous implants. There is an unmet need for developing long-acting formulations for LNG. To develop long-acting formulations, release functions need to be investigated for LNG implant. Therefore, a release model was developed and integrated into an LNG physiologically-based pharmacokinetic (PBPK) model. Utilizing a previously developed LNG PBPK model, subcutaneous administration of 150 mg LNG was implemented into the modeling framework. To mimic LNG release, ten functions incorporating formulation-specific mechanisms were explored. Release kinetic parameters and bioavailability were optimized using Jadelle^®^ clinical trial data (n = 321) and verified using two additional clinical trials (n = 216). The First-order release and Biexponential release models showed the best fit with observed data, the adjusted R-squared (R^2^) value is 0.9170. The maximum released amount is approximately 50% of the loaded dose and the release rate is 0.0009 per day. The Biexponential model also showed good agreement with the data (adjusted R^2^ = 0.9113). Both models could recapitulate observed plasma concentrations after integration into the PBPK simulations. First-order and Biexponential release functionality may be useful in modeling subcutaneous LNG implants. The developed model captures central tendency of the observed data as well as variability of release kinetics. Future work focuses on incorporating various clinical scenarios into model simulations, including drug-drug interactions and a range of BMIs.

## 1. Introduction

Long-acting formulations (LAF) are potentially well-suited drug delivery systems for drugs whose clinical action(s) require sustained systemic release over long periods of time. This includes drug classes such as antipsychotics, chronic neurodegenerative disease drugs, drugs with high potential for abuse, and hormonal contraceptives, among others. The main goal of LAF is to decrease dosing frequency while maintaining more consistent drug levels throughout the therapy duration. As a result of these changes, patients can benefit from better adherence due to the simplified regimen, as well as increases in efficacy due to more consistent drug levels. However, the clinical development of LAF is lengthy and costly. Cognizant of the challenges and limitations related to the development of LAF, the FDA has advocated the utilization of new tools and approaches for linking pharmaceutical quality to clinical performance in order to speed access to safe and effective innovative formulations and generic drug products to the public and reduce the costs to industry. In this context, model-integrated evidence approaches have emerged as scientifically sound alternatives [1].

Even though brand-name products formulated as long-acting implants containing progestin-based hormonal contraceptives have been in the US market since 1990 (Norplant^®^), no generic products have been approved yet [2]. Throughout the more recent years, other iterations of contraceptive LAF have been developed; however, issues surrounding their longer development time and costs prevent wider expansions of this type of formulation. In case of progestin-based hormonal contraceptives, formulations are typically implanted under the skin and remain there for 3–5 years which warrants very lengthy clinical trials for dose-finding and regulatory approval, thus greatly increasing development costs. Furthermore, LAF typically result in lower mean drug levels compared to their oral counterparts, which on one hand can contribute to increased safety, but also potentially risk decreased efficacy, especially when drug levels are acted upon by outside forces such as induction drug-drug interactions (DDI). For example, average steady-state concentrations (Cavg) for oral levonorgestrel are approximately 744 ng/mL, while Cavg for LAF of levonorgestrel are approximately 350 ng/mL [3,4].

The minimal clinical information on the mechanisms and characterization of the pharmacokinetics (PK) in LAF suggests the potential utility of model-based tools to establish in vitro-in vivo correlations (IVIVC) for such formulations. In other words, the promise of LAF motivates the development of mechanistic and physiologically relevant models that accurately predict systemic exposure, which can be applied to bioequivalence assessments. These integrated mechanistic models and methods for testing bioequivalence must be developed, verified and implemented to generate maximum regulatory flexibility and increase LAF product development. 

Model-integrated evidence approaches could address both efficacy concerns while also expediting LAF development by reducing development timelines and sample sizes [5]. However, in order to successfully implement such quantitative pharmacology methods, advances in the mechanistic understanding of the drug- and the formulation-specific properties must be accomplished. 

Levonorgestrel (LNG) is a common contraceptive medication used throughout the world and available in multiple formulations, including both oral and long-acting implant (Jadelle^®^). Furthermore, LNG has an approved LAF generic outside of the US, i.e., Sino-implant, which can be leveraged to explore and validate our model-integrated evidence approach for LNG’s LAF. Thus, using LNG as a prototypical contraceptive drug, we aimed to explore the extrapolation of LAF development approaches using a model integration approach. This work presents the development and validation of a release function and absorption model for LNG implants, accounting for formulation-specific parameters, i.e., drug release from device. We then integrated these models and formulation properties into a previously developed physiologically-based pharmacokinetic (PBPK) model of LNG for the purposes of LAF extrapolation and development.

## 2. Materials and Methods

In this research we applied a stepwise model-based approach. First, a previously developed PBPK modeling framework for LNG was leveraged [6]. For the second step, a model describing the release rate of LNG from implants was developed. There is a range of modeling approaches used to capture the release from a range of drug formulations. Common types of drug release models include a range of complexity from the simple Zero- and First-order release to the following more complex models: Higuchi, Korsmeyer-Peppas, Hixson Crowell, Baker-Lonsdale, Weibull, Hopfenberg, and Gompertz [7]. Given the unique considerations involved in LAF, multiple release models were tested over this range of complexity for LNG implants. Once a final release model was selected via statistical analysis, the last step required integration of the selected drug release function into the PBPK framework to represent a subcutaneous LNG implant. The final subcutaneous PBPK model was verified utilizing clinical trial data not used for model development.

### 2.1. Physiologically-Based Disposition Model

A PBPK model of LNG has been previously developed in PK-Sim^®^ v9 and was used as the base model for this project [6]. The model was able to capture central tendency as well as interindividual variability of LNG plasma concentration profiles. Additionally, the model was verified with clinical DDI studies to ensure robust applicability. Further model development with respect to a custom administration compartment within subcutaneous interstitial space and integration of the aforementioned release model was conducted using MoBi^®^ v10, and the population-based simulation of this expanded model was done with PK-Sim^®^ v10.

### 2.2. Drug Release Model

**Figure 1:**
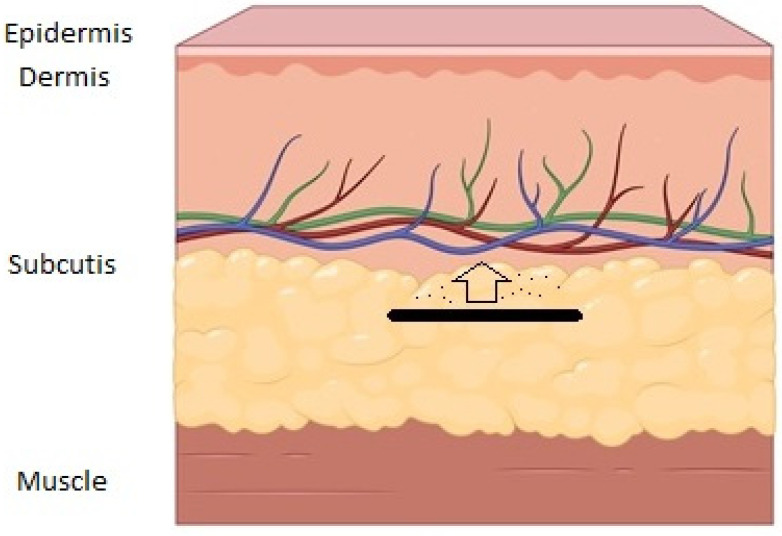
Schematic of the anatomy of the skin and where the LNG implant (black) resides after implantation along with how the drug is released and enters systemic circulation (black dots and arrow). Created with BioRender.com.

In order to capture the release kinetics of LNG from a subcutaneous implant, in vivo LNG release data were used from two publications [8,9]. To generate this data, the authors removed implant devices from study subjects at various time points to quantify the amount of LNG in the device. This was accomplished by first cleaning the removed implants, followed by LNG extraction from the device via cutting the implant, incubation in a chloroform and ethanol dilution, and then quantification via triplicate spectroscopy and HPLC for validation. A schema for where the implant device is implanted in the skin and how drug is released into systemic circulation is provided in Figure 1. Furthermore, data concerning LNG released over time from Jadelle^®^’s FDA product label were utilized to further increase confidence in the model fits [10]. These data were used to analyze cumulative LNG release over time to derive an implant input function for the PBPK simulations. The reported LNG release profiles were analyzed with ten different models. Three models are governed by the exponential function (First-order, Weibull, and Biexponential), one is a rational function (Second-order), and six are polynomial functions (Zero-order, Korsenmeyer Peppas, Higuchi, Hixon-Crowell, Peppas Sahlin, and Population Council). The mathematical equations of the models are summarized in Table 1. The model parameters were estimated by minimizing the sum of squares of the error between the data and the model curve using MATLAB routine fit and nonlinear least squares method. The key consideration of model selection was the predictive performance of the release model. The simulations were compared to the observed data and adjusted R-squared values were used to measure the goodness of fit. The physiological plausibility and the possibility of model extension are also considered. 

### 2.3. PBPK Model Integration

As previously mentioned, integration of the final release model was performed in MoBi^®^ v10. Briefly, a custom administration protocol was developed to represent an LNG 150 mg device implant. Release of LNG from the device into interstitial space and eventually systemic circulation using the selected input function was done by creating a subcutaneous depot compartment within the interstitial skin organ compartment. Based on ex vivo implant data on remaining drug levels in the implant device at the end of a five-year clinical trial, it was assumed that only a fraction of the amount of LNG in the implant device is available for release [9]. This was accounted for by using a correctional factor within the release function, indicated as (1−f), which was assumed to be 50%, or 0.50, based on available data. Simulated and observed data were compared and model adequacy was concluded if simulations are within 1.25 times of the error of respective observations. 

## 3. Results

### 3.1. Release Model Selection

Among the ten different release models tested, the First-order and Biexponential models provided a good agreement with the dataset, physiological relevance and robust parameter estimation across the different LNG devices. Thus, both models were chosen to be integrated into the LNG PBPK model. 

**Figure 2:**
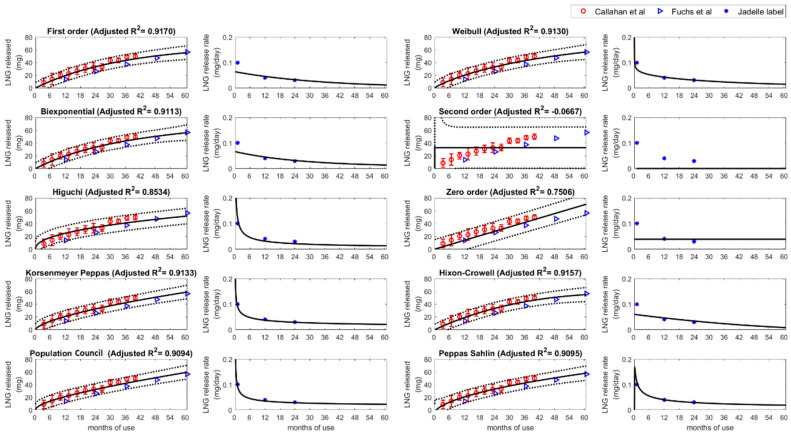
Data fitting results of ten different release models with Jadelle^®^ data. The black solid curves indicate model prediction with estimated parameters and black dotted curves represent 95% prediction intervals. The red circles represent the observed mean LNG amount with standard errors, and blue triangles show converted LNG amount from the observed LNG release rate. The first and third columns represent the LNG released amount [8,9] while the second and fourth columns show the time dependent release rate [10].

Figure 2 shows the observed data with the fitted curves for each drug release function. The First-order model showed the highest adjusted R-squared value (0.9170) followed by Hixon-Crowell (0.9157), Korsenmeyer Peppas (0.9133), Weibull (0.9130), and Biexponential function (0.9113). It is reported that the FDA-approved LNG implant, Jadelle^®^, has varying releases rates over time, e.g., 0.1 mg/day at month 1, 0.04 mg/day at 12 months, and 0.03 mg/day from 24 months on [10]. This led to the conclusion of considering a biphasic release model in addition to the best-fitting First-order model. Additionally, estimated parameters from the First-order and Biexponential models have physiological meanings; k values are release rates and the remaining amount of drug in the device could be derived from the parameter Q. 

Table 2 shows the input parameters derived from clinical data after applying different models. Full parameter set table is provided in Supplementary Table S1. $Q_{max}$ represents the maximum drug amount that can be released from the implant. In the First-order model, the parameter $Q_{max}$ is 67.9 mg, or 45.3% of original LNG load, representing the maximum amount that can be released from the implant device, which was in line with the original assumption of including an f correctional parameter value of 50% based on ex vivo data. $k_{1}$ was estimated at 0.0009 per day, representing the implant release rate. For the Biexponential model, the dose was fixed at the total amount in the implant (150 mg) with a comparable $Q_{b1}$ of 44.6 mg, and the initial release $k_{1b}$ was 0.00127 per day and a secondary release was 8.98 × 10^−5^ per day for the remaining 105 mg. The final release functions used for PBPK simulations for the first order and Biexponential functions are provided in Equations (1) and (2), respectively. Note that $Q_{max}$ is derived from the (1−f)∗Dose expression in Equation (1).

(1) $$L_{i}=\left(1-f\right)*Dose*e^{-k_{1}t_{i}}$$

(2) $$L_{i}=\left(1-f\right)*\left(\left(Q_{b1}*e^{-k_{1b}t_{i}}\right)+\left(Dose-Q_{b1}\right)*e^{-k_{2b}t_{i}}\right)$$

Sino-implant (II) data were utilized to validate our modeling approach [8]. The First-order and Biexponential models provided a good statistical performance with high adjusted R-squared values; R-squared values equal to 0.9454 and 0.9818, respectively. When comparing the estimated parameter of Jadelle^®^, the First-order model showed relatively similar parameter values. The $Q_{max}$ was 75.6 and 67.9 mg (50.4% and 45.3% of loaded LNG) and $k_{1}$ was 0.0008 and 0.0009 per day for Sino-implant (II) and Jadelle^®^, respectively. The estimated parameters for Biexponential model for Sino-implant (II) are follows: 14.95 mg for $Q_{b1}$, 0.0062 for $k_{1b}$ and 0.0002 for $k_{2b}$. It can be interpreted as, only 9.97% of loaded dose is released for approximately 5 months (1/0.0062 days), 90.03% are released relatively slower for entire insertion period (with 0.0002 per day). The data-fitting results for Sino-implant (II) release are provided in Supplementary Materials.

### 3.2. Integration into LNG PBPK Model

**Figure 3:**
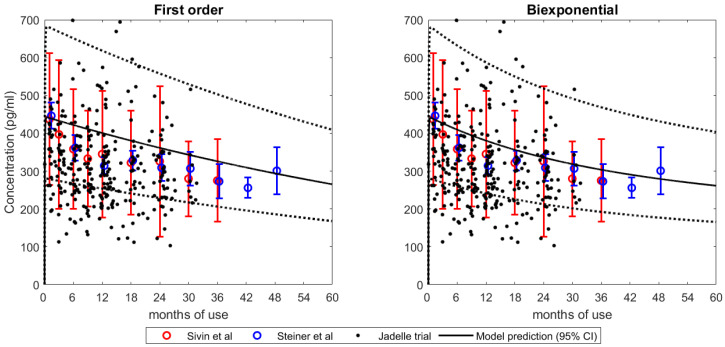
Predicted and observed in vivo concentration vs. time profiles of the PBPK model simulations with the respective release model integration. The black solid curves indicate model prediction with estimated parameters and black dotted curves represent 95% prediction intervals [11,12,13].

Figure 3 shows the plasma concentration and model predictive curve using the First-order and Biexponential release kinetics. Both models were able to capture the mean and interindividual variability, as shown by the 95% confidence interval of LNG systemic exposure. It is noteworthy that the Sivin et al. and Steiner et al. data were not utilized for model development purposes. Furthermore, predicted vs. observed Cmax and AUC ratios at the last time point for the First-order model were 0.98 and 0.96, respectively, while they were 0.98 and 1.02 for the Biexponential model, respectively.

## 4. Discussion

Our results suggest that the monophasic and biphasic release functionality may be useful in modeling LNG release from subcutaneous implants. The developed models, with the exception of the Second-order model, capture the central tendency of the observed data as well as variability of release kinetics. However, polynomial function models (Korsenmeyer Peppas, Higuchi, Zero order, Hixson-Crowell, Peppas Sahlin, and Population Council), which have no physical upper bound, are likely not appropriate for LAF development purposes. This is due to the fact that these models are monotonically increasing functions as time increases, and thus, are unable to reflect a physical maximum value required for implant devices released over long time periods. 

The two release functions utilized for PBPK simulations, i.e., First-order and Biexponential models, were selected based on both model fitting and physiological/mechanical plausibility, respectively. To clarify, while the First-order release model had the highest R^2^ value for data fit, it is known that Jadelle^®^ follows a biphasic release which motivated the selection of integrating the Biexponential release model in the PBPK simulations as well. To ensure robustness of the results, the same release modeling approach was applied to describe data from a bioequivalent formulation namely, Sino-implant (II), with good performance, providing further confidence in our modeling efforts (Figure S1). It is noteworthy that Jadelle^®^ and Sino-implant were found to be comparable in terms of PK during the first year, but slowly differentiated during the 2–4 years period [12]. Nonetheless, retrospective studies have supported the WHO notion that the two products are comparable for the first three years post-implant [10,12].

One of the challenges in the modeling and simulation process is the availability and collection of data over a sufficient length of time to adequately inform parameters. The reliability of the estimated parameters is directly dependent on the available data to ensure complete characterization of the process being modeled. In this regard, the First-order model has fewer parameters, making it an easier and more stable target for optimization with less data. However, the Biexponential model captures the mechanistic release more directly but contains additional parameters that would require additional data in order to accurately and consistently estimate all model parameters. With the full dataset, i.e., 5 years, both models had good predictions of AUC and Cmax. Additional exploration into how much and what type of data is necessary for LAF PBPK model development may help inform an integrated model that may require less extensive clinical trials for testing in bioequivalent designs. In this regard, we believe that this study lays the groundwork for which types of models may support future bioequivalence assessments of LAF.

Applying this model-integrated evidence framework can help better capture a range of expected real-world scenarios observed in contraceptives, such as drug-drug interaction scenarios and a range of BMIs. Applying model-based meta-analysis (MBMA) may help capture the changes in LNG efficacy with a more mechanism-based rationale than previously understood. Furthermore, if applied in a large range of simulations, the approach of looking at truncation of the data may facilitate more rigorous analysis regarding bioequivalence and the minimal but sufficient data that must be collected to inform the robust assessment of release. Thus, informing the minimum data needed to establish bioequivalence aids in clinical trial design and potentially reduces time-to-market.

## 5. Conclusions

This study assessed the performance of different kinetic functions to describe the release of LNG from LAF and be used as an input function into a previously defined PBPK modeling framework. The First-order and Biexponential release functions captured both the mean and variability of available clinical data. Further, both models provided similar release kinetic parameters for Jadelle^®^ and Sino-implant (II), which are bioequivalent products. In the future, the PBPK application presented in this work will be explored for clinically diverse populations such as DDI and altered BMI scenarios.

## Figures and Tables

**Table 1:** Release Kinetics Models.

Model	Released Amount at Time t	Release Rate at Time t (Released Amount per a Day)	Specific Cases	Range
Weibull	$Q=Q_{max}\left(1-e^{-{\left(k_{w}t\right)}^{l}}\right)$	$\frac{dQ}{dt}=k_{w}Q_{max}l{\left(k_{w}t\right)}^{l-1}e^{-{\left(k_{w}t\right)}^{l}}$	l = 1: First-order	$Q\in \left[0,Q_{max}\right)$
Biexponential	$Q=Dose-Q_{b1}e^{-k_{b1}t}$ $-(Dose-Q_{b1})e^{-k_{b2}t}$	$\frac{dQ}{dt}=k_{b1}Q_{e}e^{-e_{b1}t}$ $+k_{b2}(Dose-Q_{b1})e^{-k_{b2}t}$		Q∈[0,Dose)
Second-order	$Q=Q_{max}\left(1-\frac{1}{1-Q_{max}k_{2}t}\right)$	$\frac{dQ}{dt}=\frac{-Q_{max}^{2}k_{2}}{{\left(1-Q_{max}k_{2}t\right)}^{2}}$ (or $\frac{dQ}{dt}=-k_{2}{\left(Q-Q_{max}\right)}^{2}$)		$Q\in \left[0,Q_{max}\right)$
Korsenmeyer Peppas	$Q=k_{kp}t^{n}$	$\frac{dQ}{dt}=nk_{kp}t^{n-1}$	n = 0.45: Higuchi n = 1: zero-order	Q∈[0,∞)
Hixon-Crowell	$Q=Q_{hc}-{\left(Q_{hc}^{1/3}-k_{hc}t\right)}^{3}$ (or $Q_{hc}^{1/3}-{\left(Q_{hc}-Q\right)}^{\frac{1}{3}}=k_{hc}t$)	$\frac{dQ}{dt}=3k_{hc}{\left(Q_{hc}^{1/3}-k_{hc}t\right)}^{2}$		Q∈[0,∞)
Peppas Sahlin	$Q=k_{ps1}t^{m}+k_{ps2}t^{2m}$	$\frac{dQ}{dt}=mk_{ps1}t^{m-1}+2mk_{ps2}t^{2m-1}$	m = 0.5: Population Council	Q∈[0,∞)

**Table 2:** Estimated parameters for Release Models.

Model	Estimated Parameters (95% Confidence Intervals)
First-order	Dose = 150 (fixed) $Q_{max}$ = 67.86 (49.28, 86.44) $k_{1}$ = 0.0009 (0.00051, 0.0014)
Biexponential	Dose = 150 (fixed) $k_{1b}$ = 0.00127 (−0.002461, 0.005011) $k_{2b}$ = 8.985 × 10^−5^ (−0.0005871, 0.0007668) $Q_{b1}$ = 44.64 (−134.6, 223.9)

Units: Qmax (mg), Dose (mg), k (days^−1^).

## Data Availability

The final model and data of this work can be obtained via request of the corresponding author.

## Supplementary Materials

The following supporting information can be downloaded at: https://www.mdpi.com/article/10.3390/pharmaceutics15051393/s1, Table S1 lists the estimated parameter for ten drug release models. Figure S1 presents the results of the Sino-implant (II) release over time using the same release models evaluated for Jadelle^®^.
