# Supplementary material for: Model-Based Analysis of In Vivo Release Data of Levonorgestrel Implants: Projecting Long-Term Systemic Exposure
Source: Pharmaceutics. 2023 May 2;15(5):1393. doi: 10.3390/pharmaceutics15051393 (PMC10222093; doi:10.3390/pharmaceutics15051393)
Supplement: Supplementary file 1 [file pharmaceutics-15-01393-s001.zip › pharmaceutics-2301277-supplementary.pdf]

Supplemental Materials

Model-based analysis of in vivo release data of Levonorgestrel implants: projecting long-term systemic exposure

Soyoung Kim[1,*], Brian Cicali[1,*], Michelle Pressly[1], Lais Da Silva[1], Thomas Wendl[2], Valvanera Vozmediano[1], Stephan Schmidt[1,^], Rodrigo Cristofoletti[1,^]

Table S1. Estimated parameters for Mechanistic Release Models

**Model**	**Estimated parameters (95% Confidence Intervals)**
First-order	Qmax=67.86 (49.28, 86.44)<br>k1=0.0009 (0.00051, 0.0014)
Weibull	Qmax=82.65 (-11.67, 177)<br>kw=0.00065 (-0.00075, 0.0020)<br>l=0.8763 (0.408, 1.345)
Biexponential	Dose=150 (fixed)<br>k1b= 0.00127 (-0.002461, 0.005011)<br>k2b=8.985e-05 (-0.0005871, 0.0007668)<br>Qb1=44.64 (-134.6, 223.9)
Second-order	Qmax=32.67 (22.12, 43.23)<br>k2=62.11 (-1.296+7, 1.296e+7)
Korsenmeyer Peppas	kkp=0.4749 (0.05831, 0.8915)<br>n=0.642 (0.5153, 0.7687)
Higuchi	kh=1.763 (1.623, 1.903)
Zero-order	k0=0.03854 (0.03452, 0.04257)
Hixon-Crowell	khc=0.001337 (0.0009549, 0.001719)<br>Qm=57.9 (45.65, 70.16)
Peppas Sahlin	kps1=-5.176 (-35.49, 25.14)<br>kps2=2.626 (-11.32, 16.57)<br>m=0.2338 (-0.03693, 0.5046)
Population council	kpc1=0.8826 (0.5336, 1.232)<br>kpc2=0.0119 (0.00109. 0.02272)
Units: Qmax (mg), Dose (mg), k ($days^{-1}$)	

Figure S1. Results of overlaying the predicted LNG release over time using the various release models of this work with clinically observed (red) Sino-Implant (II) data. [1]

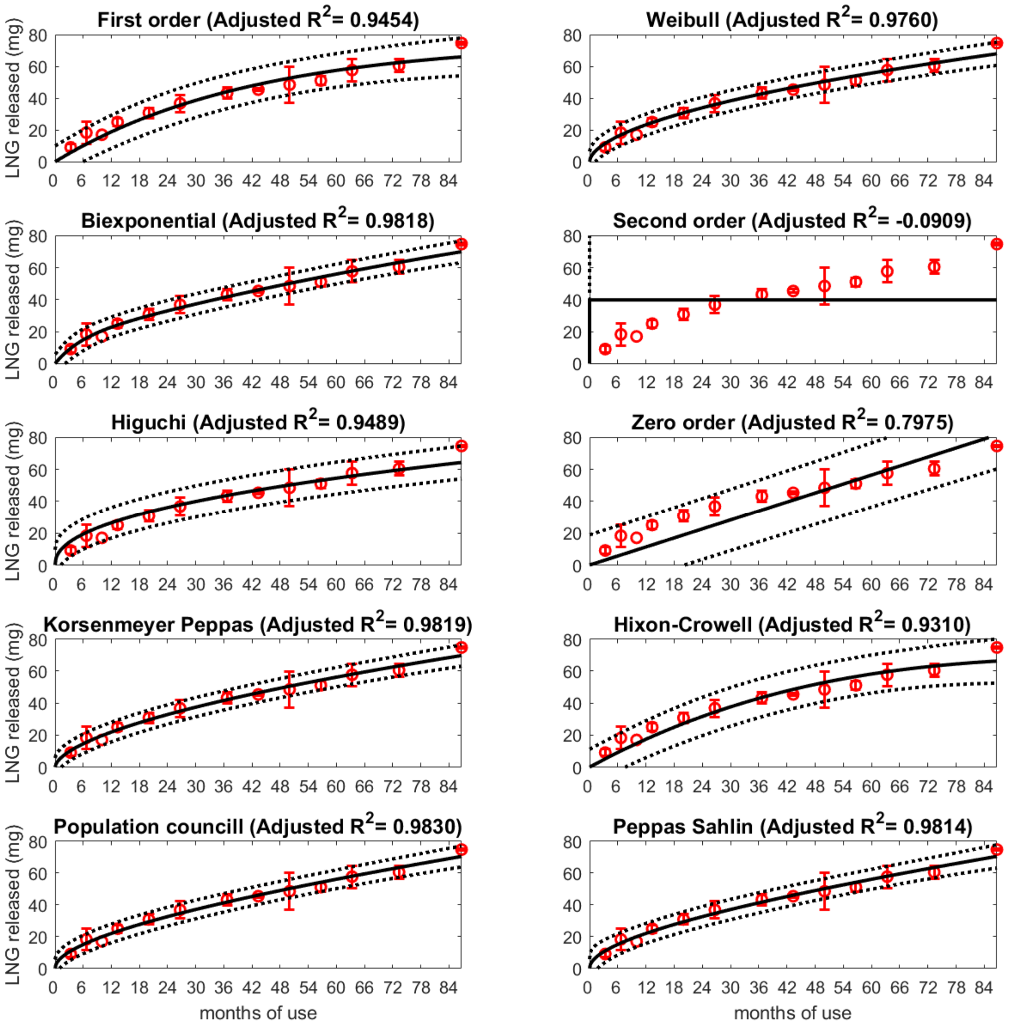

Reference

1. Callahan RL, Taylor D, Jenkins DW, Owen DH, Cheng L, Cancel AM, Dorflinger LJ, Steiner MJ. In vivo release of Levonorgestrel from Sino-implant (II) — an innovative comparison of explant data. Contraception 2015, 92:350–355
